# Apoptosis induction activity of polysaccharide from *Lentinus edodes* in H22-bearing mice through ROS-mediated mitochondrial pathway and inhibition of tubulin polymerization

**DOI:** 10.29219/fnr.v64.4364

**Published:** 2020-10-21

**Authors:** Qilin Zhang, Zhaosong Du, Yu Zhang, Ziming Zheng, Qiang Li, Kaiping Wang

**Affiliations:** 1 Department of Pharmacy, Union Hospital, Tongji Medical College, Huazhong University of Science and Technology, Wuhan, China; 2 Hubei Province Clinical Research Center for Precision Medicine for Critical Illness, Wuhan, China; 3 Department of Pharmacy, Wuhan Women and Children Medical Care Center, Wuhan, China; 4 Hubei Key Laboratory of Nature Medicinal Chemistry and Resource Evaluation, Tongji Medical College of Pharmacy, Huazhong University of Science and Technology, Wuhan, China

**Keywords:** Lentinus edodes polysaccharide, hepatocellular carcinoma, direct antitumor activity, mitochondrial apoptosis pathway, tubulin polymerization

## Abstract

**Background:**

*Lentinus edodes* is a medicinal mushroom widely used in Asian countries for protecting people against some types of cancer and other diseases.

**Objective:**

The objective of the present study was to investigate the direct antiproliferation activity and the antitumor mechanisms of water-extracted polysaccharide (WEP1) purified from *L. edodes* in H22 cells and H22-bearing mice.

**Design:**

The extraction, isolation, purification, and structure determination of the water-soluted *L. edodes* polysaccharide WEP1 were performed. The growth inhibitory effects of WEP1 on H22 cells and H22-bearing mice were determined by 3-(4,5-Dimethylthiazol-2-Yl)-2,5-Diphenyltetrazolium Bromide (MTT) method and animal studies. Flow cytometry, scanning electron microscopy, and laser scanning confocal microscopy were used to observe the morphological characteristics of apoptotic cells. The levels of intracellular reactive oxygen species (ROS) were detected by flow cytometry using 2’,7’-dichlorofluorescein-3’,6’-diacetate (DCFH-DA). Western blot was used to determine the expressions of cell cycle proteins and apoptosis-related proteins.

**Results:**

Results showed that WEP1 with a molecular weight of 662.1 kDa exhibited direct antiproliferation activity on H22 cells in a dose-dependent manner. *In vivo*, WEP1 significantly inhibited the growth of tumor at different doses (50, 100, and 200 mg/kg) and the inhibition rates were 28.27, 35.17, and 51.72%, respectively. Furthermore, morphological changes of apoptosis and ROS overproduction were observed in H22 cells by WEP1 treatment. Cell cycle assay and western blot analyses indicated that the apoptosis induction activity of WEP1 was associated with arresting cell cycle at G2/M phase and activating mitochondrial-apoptotic pathway. Besides, WEP1 disrupted the microtubule network accompanied by alteration of cellular morphology.

**Conclusion:**

Results suggested that the antitumor mechanisms of WEP1 might be related to arresting cell cycle at G2/M phase, inhibiting tubulin polymerization and inducing mitochondrial apoptosis. Therefore, WEP1 possibly could be used as a promising functional food for preventing or treating liver cancer.

## Popular scientific summary

- Polysaccharide from *Lentinus edodes* inhibited the proliferation of H22 cells and tumor growth of H22-bearing mice.
- Polysaccharide from *Lentinus edodes* induced apoptosis in hepatocellular cancer cells by arresting cell cycle at G2/M phase, increasing ROS level and Bax expression, down-regulation of Bcl-2 expression and activation of caspase-3 expression.
- The antitumor mechanisms of polysaccharide from *Lentinus edodes* might be related to inhibition of tubulin polymerization and induction of mitochondrial apoptosis.

Hepatocellular carcinoma (HCC), as a common type of malignant tumor, has become the third leading cause of cancer-related death globally (1). Traditional treatment of HCC was generally involved in surgical resection, chemotherapeutics, and radiotherapy (2). However, some patients were not eligible for resection, and the chemotherapeutics applied in clinical were not specific targets and usually had severe side effects. Therefore, searching for novel and more effective agents for prevention and targeted therapeutic of HCC without or with fewer adverse reactions is highly desirable. Increasing scientific evidences have shown that more and more anticancer drugs derived from natural products, which include thousands of functional foods and medical plants, could be applied to improve the curative effects of anticancer drugs due to their characteristics of high efficiency and low toxicity (3–5).

*Lentinus edodes*, a famous edible mushroom in our daily diet, commonly used for protecting people against some types of cancer and other diseases, such as diabetes (6) and obesity (7), has rapidly become a highly valued species for improving our health and nutrition. As one of the most effective bioactive components of *L. edodes*, polysaccharides have been proved to possess a wide variety of pharmacological properties, such as antitumor, antiviral, antioxidation, hepatoprotection, and immunomoldulating activities, especially in the aspect of antitumor effect via enhancing immunity and inducing cell apoptosis (8). *L. edodes* polysaccharides were often in combination with chemotherapeutic drugs like cisplatinum, oxaliplatin, or 5-fluorouracil to enhance the therapeutic effects and lower the dosage to reduce chemotherapy toxicity in patients simultaneously because of their advantages of comparatively non-toxic and few side effects (9, 10). However, little attention was focused on the direct killing effects of mushroom polysaccharides alone on HCC cells and the potential anticancer mechanisms. Hence, figuring out what signal pathways involved in mushroom polysaccharide-induced antitumor activity was of great significance for anticancer therapy (11).

Our previous study isolated different types of polysaccharides from *L. edodes*, which came from different places of origin and compared their structures and antitumor effects *in vitro* (12, 13). Our results fortunately found that mushroom polysaccharide has a direct antitumor effect through inducing apoptosis and inhibiting the antiproliferation of various cancer cells, such as human HCC HepG2, SMMC-7721 cells, and human breast cancer MCF-7 cells (12, 14). However, the potential molecular mechanisms of *L. edodes* polysaccharide-induced HCC cells apoptosis were still unelucidated clearly and need to explore.

Apoptosis is the process of programmed cell death and may result in abnormal expressions of apoptosis proteins and a series of morphological changes comprising membrane blebbing, cell shrinkage, nuclear fragmentation, and formation of apoptotic bodies (15). Inhibition of apoptosis was one of the distinctive features of cancer. Many natural active polysaccharides extracted from traditional plant foods such as *Dendrobium*, *Ganoderma applanatum*, and *Pleurotus nebrodensis* could induce tumor cells apoptosis through mitochondrial-mediated signaling pathways (16–18). Therefore, inducing tumor cells apoptosis was an effective strategy for cancer therapy. The structure of polysaccharide was diverse in terms of their place of origin, monosaccharide composition, main chain, and glucosidic bond (19). Therefore, in the present study, another water-soluble polysaccharide, water-extracted polysaccharide (WEP1), was extracted, purified, and preliminarily characterized from the fruit bodies of *L. edodes* that from Fangxian, Hubei Province, which was different from Zhejiang and Henan Provinces before. In particular, the apoptosis and direct antiproliferation effects of WEP1 on H22 cells, and the involved molecular mechanisms were investigated both *in vivo* and *in vitro*. The results will provide further evidences for promising use of WEP1 in the therapy of HCC, and it would be helpful to develop natural antitumor drugs from plant foods.

## Materials and methods

### Materials and reagents

Dried fruit body of *L. edodes* was obtained from Fangxian, Hubei Province, China (identified by Professor Jia-chun Chen of the Department of Traditional Chinese Medicine, Tongji Medical College of Huazhong University of Science and Technology). Anti-mouse β-actin antibody, horseradish peroxidase (HRP)-conjugated anti-mouse, and anti-rabbit secondary antibodies were provided by Sigma (St. Louis, MO, USA). Primary antibodies against Bcl-2, Bax, p53, cleaved caspase-3, p-p38MAPK, cyclin B1, cyclin D1, and CDK4 were obtained from Cell Signaling Technology (Danvers, MA, USA). Bicinchoninic acid (BCA) protein assay kit and reactive oxygen species (ROS) detection kit were purchased from Beyotime Biotechnology (Shanghai, China). Annexin V-FITC apoptosis detection kit was purchased from KeyGEN Biotech (Nanjing, China). All other reagents and chemicals were of analytical grade in this study.

### Preparation and structure determination of WEP1

**Fig. 1:**
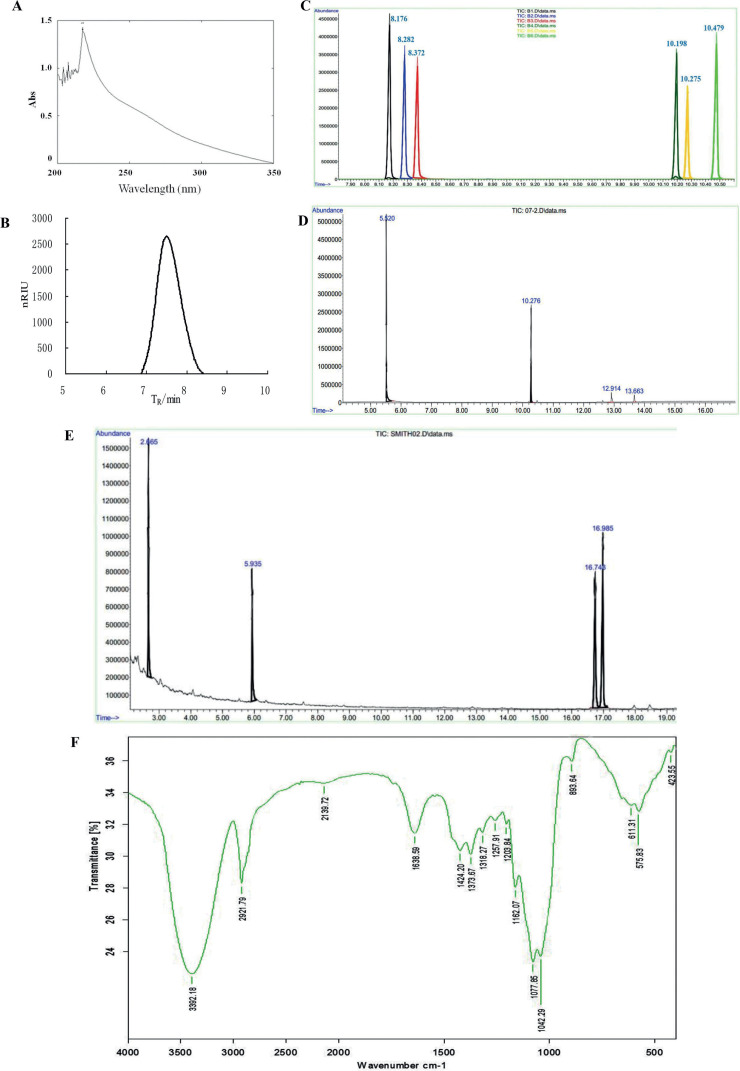
Determination of purity and chemical analyses of WEP1. (A) UV spectrum of WEP1. (B) Chromatogram of WEP1 for molecular weight determination. (C) GC of standard monosaccharides’ derivatives. (D) GC of WEP1’s derivatives. (E) GC of smith degradation for WEP1. (F) FT-IR spectrum of WEP1 in the range of 4,000–400 cm^−1^.

The extraction, isolation, purification, and structure determination of the water-soluted *L. edodes* polysaccharide WEP1 were performed as previously described (12). The content of total polysaccharide was about 98.16%. WEP1 had no absorption at 260 or 280 nm using UV scanning, indicating the absence of nucleic acids and proteins (Fig. 1A). Based on the results of gas chromatography (GC) and high-performance gel permeation chromatography (HPGPC), WEP1 was found to consist mainly of glucose (Fig. 1C, D) and to have an average molecular weight of 662.1 kDa approximately. The chromatogram of HPGPC (Fig. 1B) displayed a single and symmetrically sharp peak, demonstrating that WEP1 was homogeneous. Periodate oxidation showed (1→3)-linkage or (1→3,6)-linkage and (1→)-linkage or (1→6)-linkage existed in the molecule, and the molar ratio was about 5:3. After Smith degradation, glucose and glycerin were found in GC analysis (Fig. 1E). The presence of glucose (retention time: 16.743 and 16.985) demonstrated the existence of (1→3)-linkage or (1→3,6)-linkage. Similarly, the identification of glycerin (retention time: 2.665) showed that (1→)-linked or (1→6)-linked glycosyl residues existed, which were basically consistent with those of periodate oxidation. The fourier transform infrared (FT-IR) spectrum (Fig. 1F) of WEP1 further confirmed that WEP1 was a polysaccharide containing β-d-glucans with pyranose rings.

Compared with our previous studies, we found that the sugar content, molecular weight, monosaccharide composition, and the glycosidic bond connecting types of *L. edodes* polysaccharides obtained with the same extraction and purification method did not have much of difference (12, 13). They just have slight difference on the proportion of glycosidic bond links even though the source and producing area of *L. edodes* are differ. Results indicated that the extraction technology of *L. edodes* polysaccharide was stable, and WEP1 could be further used in the subsequent research.

### Cell culture

H22 hepatoma cell lines were provided by Tongji Medical College and cultured in Roswell Park Memorial Institute (RPMI)-1640 medium with 10% fetal bovine serum, 100 U/mL penicillin, and 100 mg/mL streptomycin. Cells cultures were incubated in a humidified atmosphere of 5% CO_2_ at 37°C. WEP1 was dissolved in medium at different concentrations.

### Cell viability assay

The growth inhibitory effects of WEP1 on H22 cells were evaluated by MTT assay. Tumor cells (5 × 10^4^ cells/mL) in 96-well plates were treated with various concentrations of WEP1 (12.5, 25, 50, 100, 200, 400, and 800 μg/mL) in RPMI-1640 medium for 24 h before 20 μL MTT (5 mg/mL) was added to each well. After incubation for another 4 h at 37°C, the supernatant was removed, and 150 μL dimethyl sulfoxide (DMSO) was added to dissolve the MTT formazan. The plates were measured at 570 nm (*A*_570_). Cell growth inhibition ratio was calculated as follows: (1-*A*_570_ of treated cells/*A*_570_ of untreated cells) × 100%.

### Flow cytometry detection of apoptosis

Quantitative measurement of apoptosis was analyzed by double staining using Annexin V-FITC and propidium iodide (PI), as previously described (14). Briefly, H22 cells (5 × 10^4^ cells/mL) grown in 12-well plates were exposed to different concentrations of WEP1 (375, 750, and 1,500 μg/mL) for 24 h. After the treatment, the cells were washed twice in phosphate buffered solution (PBS). Then the cells were collected and resuspended in binding buffer followed by staining using Annexin V-FITC and PI at room temperature for 15 min in the dark. The apoptotic cells were analyzed by flow cytometry (BD, New Jersey, USA).

### Cell cycle analysis by flow cytometry

H22 cells (2 × 10^5^ cells/mL) were seeded into 6-well plate and exposed to various concentrations of WEP1 (375, 750, and 1,500 μg/mL) for 24 h. Cells were harvested, washed with PBS, fixed in ice-cold with 70% ethanol, and stained with PI. Cell cycle distribution was performed by flow cytometry (Becton Dickinson FACS Caliber, USA).

### Morphology observation of apoptotic cells by scanning electron microscopy

The morphological characteristics of apoptotic cells were determined by scanning electron microscopy (SEM) as previously reported by some modifications (20). H22 cells were treated with 1 mL of different concentrations of WEP1 (375, 750, and 1,500 μg/mL) for 24 h. Cells just incubated with medium served as negative control. The control and treated cells were collected by centrifugation, washed with PBS, and fixed with 2.5% (v/v) glutaraldehyde solution at 4°C overnight, and then collected again, suspended with 30 µL PBS, and seeded on coverslips, which were further fixed with 2.5% glutaraldehyde solution. Cells were dehydrated through graded ethanol (80, 85, 90, and 95%), dried, and stored at 60°C. Samples were sputter-coated with gold and observed using SEM at the magnification of 6,000 times.

### Assessment of tubulin network disruption by laser scanning confocal microscopy

H22 cells (2 × 10^5^ cells/mL) were cultured in 6-well plate and treated with different doses of WEP1 (375, 750, and 1,500 μg/mL) for 24 h. Subsequently, cells treated with or without WEP1 were washed with PBS, fixed with 2% paraformaldehyde for 20 min, and permeabilized with 0.1% Na-citrate and 0.1% Triton X100. Cells were then stained with mouse monoclonal fluorescein isothiocynate (FITC)-conjugated anti-α-tubulin antibody (1:100) and Hoechst 33258 for 1 h at 37°C. After incubation, cells were washed with PBS and observed under a Zeiss LSM 510 Meta confocal microscope (Germany). Images were processed with laser scanning confocal microscopy (LSCM) software.

### Measurement of intracellular ROS level

The levels of intracellular ROS were detected by flow cytometry using DCFH-DA. Briefly, H22 cells at a density of 5 × 10^4^ cells/mL were seeded in 12-well plates and exposed to different concentrations of WEP1 (375, 750, and 1,500 μg/mL) for 24 h. Afterwards, the cells were collected and incubated with 10 μM DCFH-DA for 30 min at 37°C, and then harvested, washed with PBS twice, and resuspended in ice-cold PBS in the dark. The intracellular intensity of fluorescence was observed under fluorescence microscope and measured with flow cytometry (BD).

### Antitumor activities in vivo

Male BALB/c mice (7–8 weeks old, weighing 20 ± 2 g) were purchased from the Animal Center of Disease Control and Prevention of Hubei Province, China, and housed under the standard conditions of temperature (22 ± 2°C), relative humidity (60 ± 5%), and 12 h light/12 h dark cycle. BALB/c mice were subcutaneously injected with 6 × 10^6^ cells/mL H22 cells and divided into five groups (*n* = 10) randomly and then subjected to intraperitoneally injected with various doses of WEP1 (50, 100, and 200 mg/kg) once a day for 15 days. Negative control group mice were intraperitoneally injected with the same volume of 0.9% normal saline (NS) only. Cyclophosphamide (CTX) dissolved in 0.9% NS (25 mg/kg) was used as positive control group. Tumor volume (mm^3^) was received by measuring the diameter with calipers on days 4, 7, 10, 13, and 16 and calculated by 1/2(*A* × *B* ^2^), where *A* and *B* represent the long and short dimensions of the tumor, respectively. Mice were sacrificed by cervical dislocation at the end of treatments, and the tumors and spleens were collected and weighed. The inhibitory rate against tumor growth was calculated as follows: inhibitory rate (%) = [(*C*−*T*)/*C*] × 100, where *C* was the average tumor weight of model control group while *T* was the average tumor of treated groups.

### Western blot analysis

Tumor tissues were lysed and homogenized with RIPA Lysis buffer. Protein concentrations were determined by BCA protein assay kit with bovine serum albumin (BSA) as standard according to the instructions. Total proteins (80 μg) were separated by 10 or 12% sodium dodecyl sulfate-polyacrylamide gel electrophoresis under reducing conditions, and then transferred onto nitrocellulose membranes (Amersham Biosciences, USA). Afterwards, membranes were blocked with 5% non-fat milk in TBST [0.1% Tween-20 in tris buffered saline (TBS) buffer] and incubated at 4°C overnight with primary antibodies, after which the membranes were washed three times and incubated with HRP-conjugated goat-anti-mouse or anti-rabbit secondary antibody. The bound antibody was measured with an ECL kit and immediately exposed to X-ray films. Protein expressions were calculated using the ratio of density of each protein to the density of β-actin or glyceraldehyde-3-phosphate dehydrogenase (GAPDH).

### Statistical analysis

All experimental data were expressed as means ± SD (standard deviation) of three independent experiments. Statistical analysis was performed with Student’s *t* test using a statistical analysis system software package (SPSS 19.0), and *p*-values of <0.05 were considered to be statistically significant.

## Results and discussion

### Antitumor effects of WEP1 on H22 cells and H22 tumor-bearing mice

**Fig. 2:**
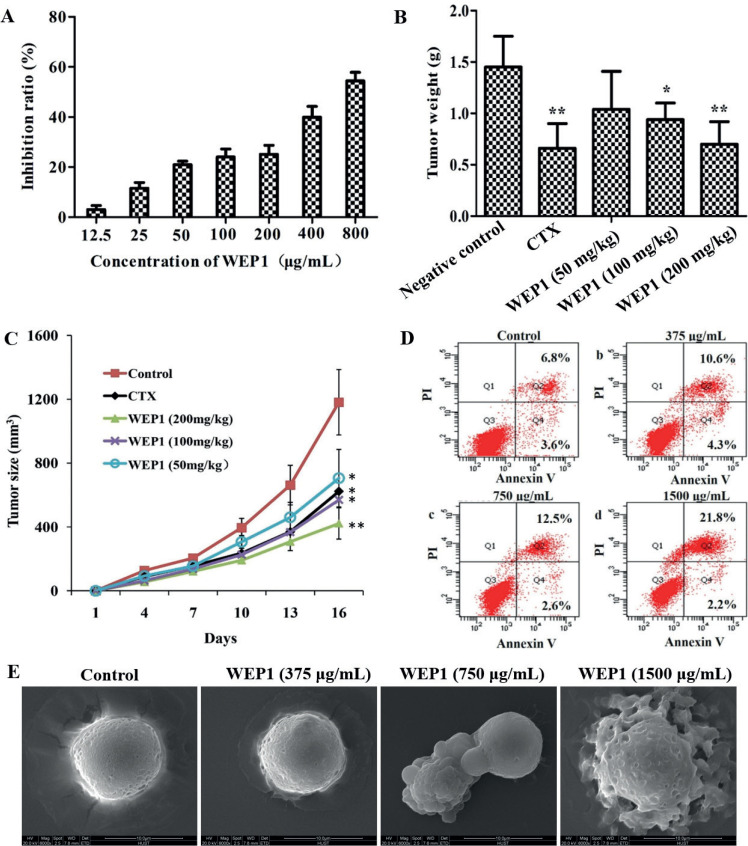
Effects of WEP1 on the growth inhibition of H22 cells and H22-bearing mice. (A) Inhibitory effect *in vitro* of WEP1 against H22 cells at 24 h treatment. (B, C) Inhibitory effect of polysaccharide WEP1 on H22 tumor growth in BALB/c mice. *P < 0.05 and **P < 0.01 compared with negative control group. (D) WEP1-induced apoptosis in H22 cells. Apoptosis was assayed by flow cytometry after cells were treated without (a, control) or with (b) 375, (c) 750, and (d) 1,500 μg/mL of WEP1 for 24 h. (E) Scanning electron micrographs (magnification ×6,000) of H22 cells showed the surface morphology changes of the cells. Data were presented as means ± SD of three independent experiments. Values were based on eight mice in each group.

Abnormal proliferation was one of the most remarkable characteristics of tumor cells. Previous researches indicated that mushroom polysaccharides could be used for cancer therapy for their direct antitumor activity. To elucidate the inhibitory effect of WEP1 on H22 cells, cells were treated with various concentrations of WEP1 (12.5, 25, 50, 100, 200, 400, and 800 μg/mL) for 24 h *in vitro*, and followed by MTT assay. As shown in Fig. 2A, WEP1 exhibited excellent direct antitumor activity against H22 cells and displayed concentration dependent inhibition activities (*P* < 0.05 or *P* < 0.01) as compared to the control. In particular, the maximum inhibition ratio of WEP1 reached 54.4%, which was comparable to other reported polysaccharides that inhibited H22 tumor growth (1, 21).

To further evaluate, if WEP1 suppresses H22 tumor growth *in vivo*, the tumor-bearing mice were injected with WEP1, CTX, and saline every day for 14 days. Compared with model control group, the tumor weights (Fig. 2B) of CTX and WEP1 groups were significantly reduced, and WEP1 efficiently inhibited the growth of tumor in a dose-dependent manner. Supporting this observation, WEP1 clearly decreased the average size of tumor volume (Fig. 2C). Results were summarized in Table 1. Collectively, results indicated that WEP1 showed obvious antitumor properties at different concentrations, with the inhibition ratios being 28.27, 35.17, and 51.72%, respectively, and the antitumor effect of the high-dose WEP1 group was comparable to those of CTX (51.72% vs. 54.48%, Table 1), which was commonly used as a chemotherapy drug in clinical. Additionally, a prominent increase of spleen index in WEP1-treated groups was observed, whereas spleen index of CTX group decreased significantly (*P* < 0.01). There were no signs of toxicity in mice treated with polysaccharide, whereas mice treated with CTX were apathetic, hardly active, and exhibited some toxicity reactions, such as reduced body weight, dim hair, and reduction in spleen index, which indicated that CTX was a double-edged sword to cancer. CTX could delay tumor progression, but it would harm immune organs at the same time. On the contrary, WEP1 exerted strong antitumor effects and immune enhancement function and therefore could be used as a potential therapeutic agent in HCC therapy. All results suggested that the antiproliferative effects of WEP1 on H22 cells were not due to toxicity but rather possibly because of its direct antitumor activity.

**Table 1:** Effects of water-extracted polysaccharide (WEP1) on spleen index, tumor volume, and tumor inhibition rate of H22-bearing mice^a^

Group	Dose (mg/kg)	Spleen index (mg/g)	Tumor volume (mm3)	Tumor weight (g)	Inhibition ratio (%)
Control	–	7.36 ± 2.31	1,181 ± 205	1.45 ± 0.30	–
Cyclophosphamide (CTX)	25	3.65 ± 0.80**	623 ± 180*	0.66 ± 0.24**	54.48
WEP1	50	9.38 ± 1.98	706 ± 180*	1.04 ± 0.37	28.27
	100	11.79 ± 1.98**	569 ± 108*	0.94 ± 0.16*	35.17
	200	14.27 ± 2.19**	422 ± 98*	0.70 ± 0.22**	51.72

a H22-bearing mice were subjected to intraperitoneally injected with different doses of WEP1 for 15 days once daily as experimental groups, and CTX was served as positive control. The tumor weight and volume were measured on the 16th day. Data are expressed as means ± SD based on eight mice for each group.

* *P* < 0.05 and **P < 0.01 as compared with control group.

### Effect of WEP1 on the apoptosis induction of H22 cells

The development of HCC involves various genetic and molecular changes in cell proliferation and resistance to apoptosis, resulting in failing to elicit the death signaling pathway. Therefore, inducing tumor cells apoptosis was an effective and promising approach for cancer therapy (22). Previous studies have reported that mushroom polysaccharides could induce apoptosis in a variety of tumor cells, including human hepatoma cells, lung carcinoma, and colon cancer (18, 23, 24). Therefore, in our study, to better prove whether the growth inhibitory effect was associated with apoptosis, a double-staining method using FITC-labeled annexin V and PI was performed. As Fig. 2D indicated, control group showed that most of the cells remained alive, while WEP1 induced significant apoptosis in H22 cells in a dose-dependent manner. The proportion of apoptotic cells significantly increased after treatment with WEP1. The percentages of apoptotic cells with 375, 750, and 1,500 μg/mL WEP1 were 14.9, 15.1, and 24.0%, respectively, which had significant differences as compared with untreated cells. Besides, the morphological characteristics of H22 cells apoptosis that induced by WEP1 were further identified by SEM. We observed that untreated cells were spherical shape and showed intact cell membrane and distinct cell borders with dense microvilli on the surface (Fig. 2E). After treatment with WEP1, significant morphological changes of apoptosis, which include loss of microvilli, decrease in cell volume, plasma membrane blebbing, and formation of wrinkles on cell surface, were observed obviously. These results were correlated with those findings of apoptosis rate and MTT. Consequently, data suggested that the antiproliferation effect of WEP1 might be associated with its potential to induce apoptosis in H22 cells.

### Effect of WEP1 on tubulin polymerization of H22 cells

**Fig. 3:**
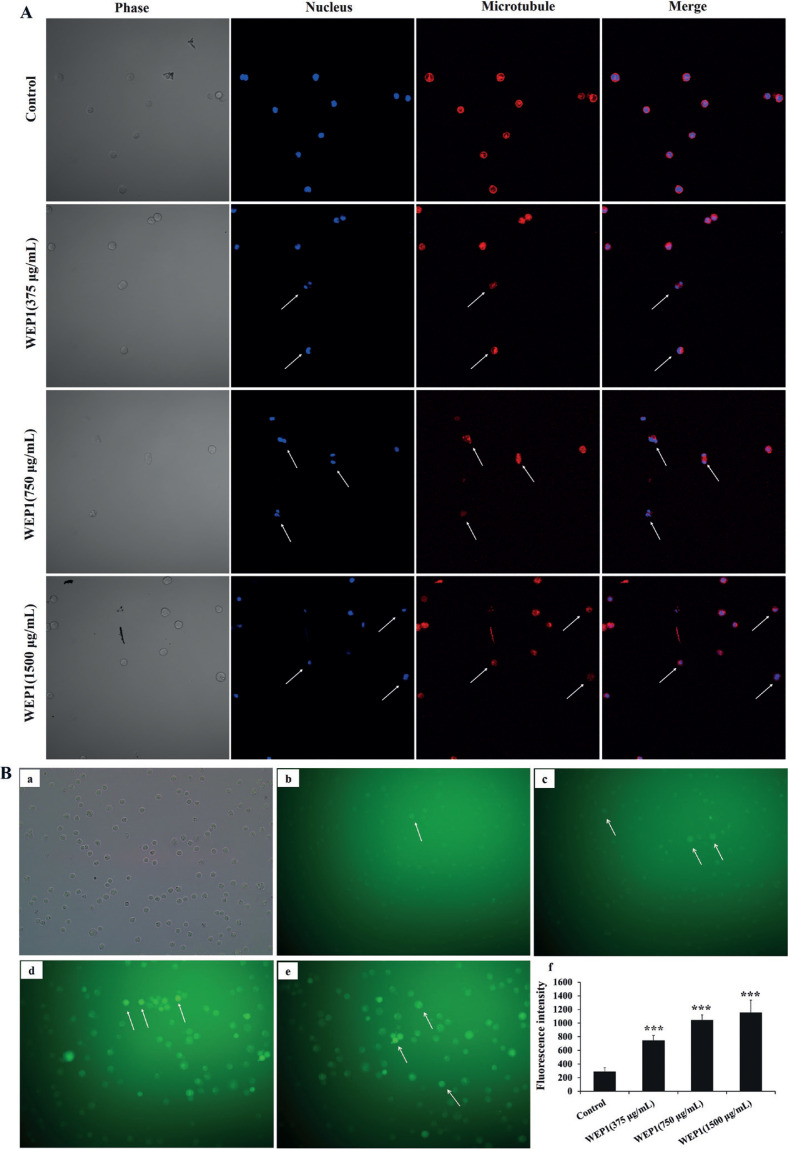
Effects of WEP1 on the inhibition of tubulin polymerization and the levels of intracellular ROS in H22 cells. (A) Disruption of microtubule network in response to WEP1-treated H22 cells was observed under LSCM. Changes were marked with arrows. (B) (a–e) Representative images of ROS fluorescence in different groups: (a) light microscope image of H22 cells; (b) control; (c–e) cells treated with different concentrations of WEP1 (375, 750, and 1,500 μg/mL). (f) Fluorescence intensity of H22 cells. Values were expressed as means ± SD from three independent experiments. ***P < 0.001 compared with control group.

It was reported that microtubules in cells were related to cell mitosis, proliferation, and apoptosis, and tubulin was one of the major structural proteins, which participated in maintaining cell integrity and cell morphology (25). In this study, to evaluate whether WEP1 altered the cellular microtubule network in H22 cells, it was examined by LSCM. As displayed in Fig. 3A, control H22 cells showed typical normally organized microtubule structure, and the microtubules were radiated outside the nucleus. However, 375 μg/mL WEP1 started to distort the microtubule network at 24 h. Middle dose of WEP1 (750 μg/mL) caused significant change of microtubule structure with reduced microtubule density at periphery, and the radial organization of microtubules was perturbed. Higher dose of WEP1 (1,500 μg/mL) drastically disrupted the central microtubule network, showing the microtubule depolymerization more obvious and resulting in an alteration of the morphology of H22 cells, which became smaller in size and rounder shaped. Results indicated that WEP1 might inhibit the cellular tubulin polymerization, depolymerize cellular microtubule, and finally induced morphological changes of apoptosis in H22 cells, which could further support results of SEM.

### Effect of WEP1 on ROS production

Excessive concentrations or the overproduction of ROS mainly produced by mitochondria could induce protein oxidation and DNA damage in cancer cells, followed by cell death or apoptosis, thus ROS generation was considered closely related to mitochondrial dysfunction and apoptosis induction of cancer cells induced by various antitumor drugs (26). Also, many polysaccharides have been demonstrated to elevate ROS levels and induce apoptosis in human cancer cells (27). Among these, *Pleurotus eryngii* polysaccharide promotes apoptosis in human hepatoma HepG2 cells through the activation of ROS-mediated pathway (28). In order to speculate whether WEP1 stimulated intracellular ROS generation in H22 cells, the fluorescent dye DCFH-DA and flow cytometer were used to calculate the accumulation of peroxides and superoxide. Quantitative analysis revealed that after exposure to 10 μM DCFH-DA for 24 h, the fluorescent cells gradually increased in varying degrees. The percentages of fluorescent cells in experimental groups were 7.8, 22.08, and 40.6%, respectively, whereas it was only 1.8% in untreated cells (data were not shown). Concomitantly, the green 7’-Dichlorofluorescein (DCF) fluorescence intensity was found to be progressively stronger when the concentration of WEP1 increased (Fig. 3B). Results showed remarkable increase of intracellular ROS by WEP1 treatment as compared to untreated cells, suggesting that WEP1 treatment might disrupt the redox balance in H22 cells and ROS might be a key mediator, which might result in H22 cells mitochondrial function damage and lead to eventual cancer cells apoptosis.

### Effect of WEP1 on the expression of p-p38MAPK in H22 tumor tissues

Growing evidence has indicated that the generation of ROS could lead to cell apoptosis accompanied with triggering the activation of mitogen activated protein kinases (MAPKs), which include extracellular regulated protein kinase (ERK), c-Jun N-terminal kinase (JNK), and p38MAPK (29). p38MAPK was regarded as one of the most important intracellular survival signal and frequently activated in diverse cancers, such as breast (30), liver (31), and lung (32) cancers, suggesting its role in cancer progression. Generally, suppression of p38MAPK pathway could result in cell cycle arrest, which was commonly regulated by cyclins (cyclin D1 and cyclin B1) and tumor suppressor protein p53. A related study reported that treatment with barbaloin induced apoptosis and G2/M cell cycle arrest in A549 cells. Moreover, barbaloin suppressed the invasion and migration of A549 cells by the inactivation of p38MAPK pathway (32). Therefore, inhibition of p38MAPK pathway might be effective approaches for the treatment of cancers, including hepatocellular cancer. In this study, we evaluated the expression of the important member p-p38MAPK in H22 tumor tissues by western blotting. Results revealed that the expressions of p-p38MAPK were sharply down-regulated by 36, 34, and 36% at the dosage of 50, 100, and 200 mg/kg, respectively with WEP1 treatment (Fig. 5A), demonstrating that p38MAPK pathway was probably a target of WEP1, and WEP1 could inhibit p38MAPK pathway to induce apoptosis.

**Fig. 4:**
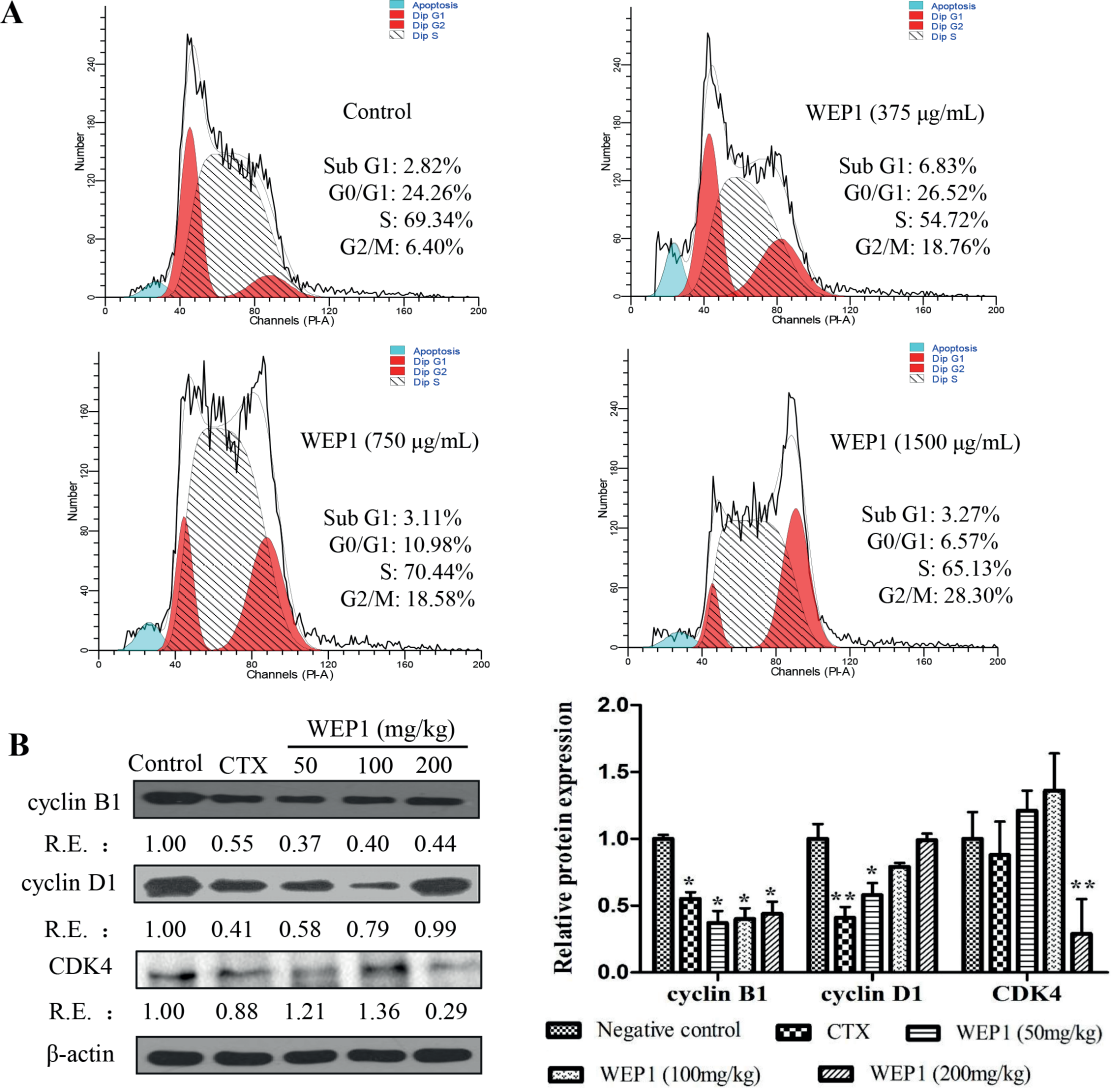
(A) Analysis of cell cycle distribution of H22 cells by flow cytometer. Cultured H22 cells were treated with 375, 750, and 1,500 μg/mL WEP1 for 24 h. (B) Effects of WEP1 on the expressions of cell cycle proteins in H22 tumor tissues. The relative ratio was expressed as a relative expression (RE) value calculated from the ratio of target protein to β-actin protein. Data represented three independent experiments. *P < 0.05 and **P < 0.01 compared with negative control group.

### WEP1 induced cell cycle arrest at G2/M phase

Cell cycle arrest could be a vital drug target to prevent uncontrolled cell proliferation and eventually induce tumor cells apoptosis. A water-soluble polysaccharide from *Poria cocos* was shown to have growth-inhibitory effects on MCF-7 cells mediated by cell cycle arrest and apoptosis induction (33). In this study, flow cytometer was used to quantify the induction of apoptosis and the effect on H22 cell cycle progression by WEP1. As shown in Fig. 4A, treatment with WEP1 at the middle and higher concentrations (750 and 1,500 μg/mL) decreased the percentage of H22 cells in G0/G1 phase from 24.26% of the untreated cells to 10.98 and 6.57%. In addition, WEP1 significantly caused accumulation of 18.76, 18.58, and 28.30% cells at different concentrations (375, 750, and 1,500 μg/mL), respectively, in G2/M phase compared with 6.40% cells in the control group (Fig. 4A). Results clearly indicated that the inhibition of H22 cells growth by WEP1 treatment was probably, at least partly, due to arresting cell cycle at G2/M phase. Further confirming the result, we detected the expressions of cell cycle proteins in H22-bearing mice. As illustrated in Fig. 4B, cyclin B1 was markedly down-regulated, and the inhibitory actions of WEP1 on cyclin B1 were 63, 60 and 56%, respectively, compared with the control. However, little changes in cyclin D1 and CDK4 expressions were observed after the treatment. Previous researches reported that cyclin D1 and CDK4 controlled the cell cycle progression of G1/S phase, whereas cyclin B1 controlled G2/M phase (34, 35). These outcomes suggested that polysaccharide WEP1 arrested cell cycle in G2/M phase through down-regulation of cyclin B1 expression in H22 tumor-bearing mice, which was consistent with results of flow cytometry, but it seemed to be different from other anticancer drugs. It was reported that different antitumor drugs had selectivity on different cell proliferation cycles. For example, polysaccharide from *P. nebrodensis* could inhibit the proliferation of human hepatoma carcinoma HepG2 cells by arresting the cell cycle at G0/G1 phase (18), while *Lycium barbarum* polysaccharide could arrest H22 cells at the S phase directly (21). Besides, the cell cycle arrest was also strongly associated with inhibition of tubulin polymerization. Combining with the results of the cellular microtubule morphology research on H22 cells, we were encouraged to conclude that WEP1 possibly arrested H22 cells in G2/M phase to induce apoptosis by inhibiting tubulin polymerization.

**Fig. 5:**
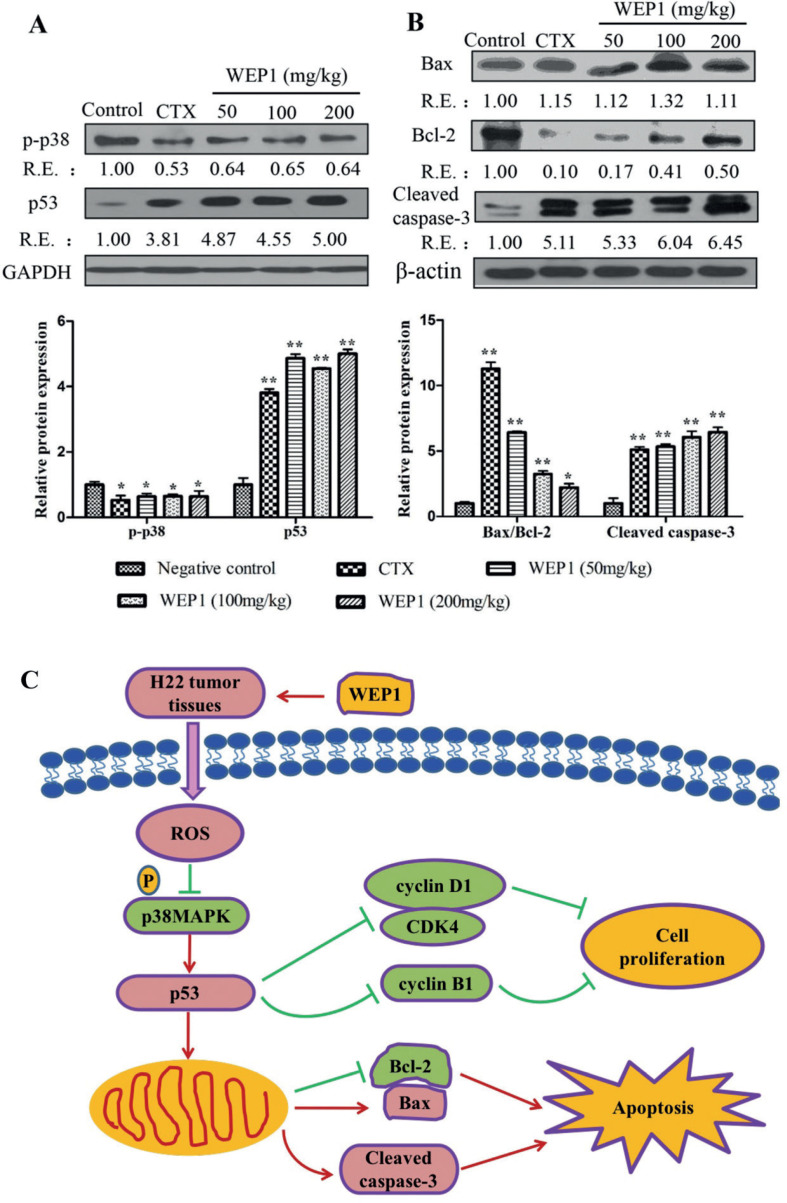
(A, B) Western blot analyses of apoptosis-related protein expressions by WEP1 treatment in H22 transplanted tumors. The relative ratio was expressed as a relative expression (RE) value calculated from the ratio of target protein to β-actin or GAPDH protein. Data represented three independent experiments. *P < 0.05 and **P < 0.01 compared with negative control group. (C) Potential mechanism of actions of WEP1-regulated cell proliferation and apoptosis in H22-bearing mice through ROS-mediated mitochondrial pathway.

### Effect of WEP1 on the expressions of apoptosis-related proteins

It was well known that apoptosis was triggered by two different death signaling pathways, including the extrinsic pathway and the intrinsic pathway. The extrinsic pathway generally initiated with the activation of death receptors by death ligands, such as tumor necrosis factor α and the Fas ligand. However, the intrinsic mitochondrial-dependent pathway involved the disruption of outer mitochondrial membrane integrity, leading to changes of the expressions of several mitochondrial genes, such as Bax, Bcl-2, and p53. The event would result in the release of cytochrome *c* from the mitochondrial intermembrane space into the cytosol, and then activate caspase-3, resulting in apoptosis (11, 36). Our study revealed that elevated ROS might lead to oxidative damage to mitochondria and finally induced cell apoptosis. As a result, to further verify the mechanisms by which WEP1 induced H22 cells apoptosis, we investigated the levels of apoptosis-related proteins, including p53, Bcl-2, Bax, and cleaved caspase-3. Western blot analyses of p53 showed nearly 3.87, 4.55, and 4.00 folds increases in WEP1 treatment group at different concentrations (Fig. 5A), suggesting that accumulated p53 was an important activator for the intrinsic mitochondrial apoptosis pathway. Besides, Bcl-2 expressions were reduced about 83, 59, and 50% at various doses (Fig. 5B) after treatment with WEP1 compared with untreated groups, whereas the distinct increase in Bax level was 12, 32, and 11% at different doses. The ratios of Bax/Bcl-2 almost increased 5.42, 2.23, and 1.21 folds (Fig. 5B) at 50, 100, and 200 mg/kg, respectively. Hence, it seemed that WEP1-induced apoptosis was correlated with elevation in p53 level and concomitant changes of Bax/Bcl-2 ratio, which was crucial for the activation of the mitochondrial-apoptotic pathway. Besides, p38MAPK was a cell survival signal that stimulated Bcl-2 protein expression, and increased expression of Bcl-2 in endothelial cells. These evidences strongly suggested that the attenuation of p38MAPK by WEP1 might be responsible for the reduced expression of Bcl-2 (37). In addition to reduced expression of Bcl-2 in apoptosis, p53 was also reported to be capable of directly activating Bax at the protein level. In this study, our data have shown that the expression of antiapoptotic protein Bcl-2 was reduced, whereas the proapoptotic protein Bax was increased in H22 tumor tissues by WEP1 treatment. The resulting increase of the Bax/Bcl-2 ratio was in accordance with previous reports on cancer cell apoptosis induced by other polysaccharides (5).

Growing evidences showed that the cascade of apoptosis-related caspases would be activated and finally lead to cell apoptosis. Among them, the most key apoptosis executioner was cleaved caspase-3 (38). Thus, the underlying expression of cleaved capspase-3 in H22 tumor tissues by WEP1 treatment was further measured by western blot. In our study, marked increases of cleaved caspase-3 were detected in comparison with the negative control after treatment with WEP1. The levels of cleaved caspase-3 markedly increased about 4.33, 5.04, and 5.45 folds at various doses (Fig. 5B), respectively. Accordingly, these occurrences further confirmed that caspase-3 activation was relevant to WEP1-induced mitochondrial-mediated apoptosis in H22-bearing mice.

## Conclusion

In summary, our present study showed a great antitumor effect of WEP1 on H22-bearing mice, reaching up to 51.72% with much lower cytotoxicity. WEP1 could suppress proliferation and induce apoptosis in H22 cells and H22 tumor-bearing mice in a dose-dependent manner. The induction of apoptosis was closely associated with ROS production, reduced p-p38MAPK expression, and simultaneously activated mitochondrion-mediated apoptotic pathway. Besides, WEP1 arrested cell cycle at G2/M phase by inhibiting tubulin polymerization and disrupting the microtubule network, and finally resulted in cell apoptosis. These findings implied that p38MAPK signaling pathway might, at least partly, be involved in the antitumor effects of WEP1. The possible mechanism of apoptosis induction by polysaccharide of this study was illustrated in Fig. 5C. This study clarified a previously unknown mechanism for WEP1-induced hepatocellular cancer cells apoptosis. Based on our results, we concluded that WEP1 from *L. edodes* had a potential apoptosis induction activity on tumor cells, and it could be regarded as an ideal candidate or a nutritional food for therapeutics of HCC.
